# Challenge-obstacle stressors and cyberloafing among higher vocational education students: the moderating role of smartphone addiction and Maladaptive

**DOI:** 10.3389/fpsyg.2024.1358634

**Published:** 2024-04-18

**Authors:** Emilio Jesús Lizarte Simón, Meriem Khaled Gijón, María Carmen Galván Malagón, José Gijón Puerta

**Affiliations:** ^1^Department of Didactics and School Organization, University of Granada, Granada, Spain; ^2^Laboratory for Cognition, Health, Training and Interaction Among Humans, Animals and Machines, University of Granada, Granada, Spain; ^3^Department of English Philology, University of Extremadura, Badajoz, Spain

**Keywords:** cyberloafing, challenge, hindrance, stress, smartphone addiction

## Abstract

The start of higher vocational education and training is a new stage for students with a challenge between theoretical classes and the new expectations and demands of companies during the internship period. To understand some of the implications of stress on cyberloafing, we can distinguish between stress perceived as an obstacle that can be overcome – challenge stress – or as a threat that can block work performance – obstacle stress – and stress perceived as an obstacle that can be overcome – challenge stress – or as a threat that can block work performance – obstacle stress-. The aim of this research is to find out the relationships between challenge-obstacle stress in Cyberloafing, as well as the moderating effect of Smartphone Addiction and Maladaptive. In this study, the Challenge-Hindrance Stressors, Smartphone addiction scale-short version (SAS-SV) instrument, the Maladaptive subscale of the Cognitive Emotional Regulation Questionnaire (CERQ) and Cyberloafing were applied to 403 upper-level vocational training students from different secondary schools in all provinces of the autonomous community of Andalusia, Spain, distributed throughout the provinces that make up this autonomous community. The findings show that students’ challenge stressors do not increase Cyberloafing, enabling them to cope with the academic demands and work challenges during the theory and internship period. On the contrary, obstacle stressors generate stressful situations that undermine the acquisition of objectives and development of academic competences. In our research we observe that challenge-obstacle stressors have a disparate influence on cyberloafing. Challenge stressors are negatively related to Maladaptive. The same is not true for obstacle stressors.

## 1 Introduction

In recent decades, vocational education and training has been seen as a type of education that, through training for the performance of a professional activity, can help to meet the challenges of digitalization and globalization (Bürgi and Gonon, 2021). According to the OECD (2018) Getting Skills Right: Spain reports on the State of the Education System of the Spanish Ministry of Education and Vocational Training, the number of jobs generated by digitalization and the ecological transition will be the two major transforming elements of the economic model (Ministerio de Educación y Formación Profesional (MEFP), 2021; Ley Orgánica, 2022). These jobs will need to be filled by competent and professionally qualified people, through training scenarios that connect theoretical knowledge with practical skills and vice versa. Forecasts for Spain in 2025 indicate that 49% of jobs will require intermediate skills and only 14% of jobs will require low skills.

The vocational education and training system has had to be transformed in order to train students through the development of professional competences. These competences facilitate entry into the world of work, which today is characterized by continuous change (Virgós et al., 2022). For this changing world, vocational education is shaped by the rationality of school and the rationality of workplace-based learning (Schaap et al., 2012). The former is related to theoretical knowledge, while the latter is related to practical knowledge (Ferm, 2021). Both knowledge is essential for the development of professional competences (Bouw et al., 2021) and, for this development, a link between theory and practice is required. Theoretical knowledge carries more weight in the field of education, while practical knowledge is more important in the work context (Billett, 2014). In both contexts, however, the use of ICT-supported communication platforms appears in relation to productivity and the challenges of digitalization. This incorporation has changed contemporary organizations (Tandon et al., 2021) and, since its beginning, there has been debate about the benefits and drawbacks that new technologies can bring in relation to their productive or non-productive use during working hours. Thus, we enter into the issue of time spent using technologies for activities that bring personal benefit unrelated to work performance (Batabyal and Bhal, 2020; Tandon et al., 2021), even in the case of students in academic training. This behavior is referred to as Cyberloafing (Lim, 2002; Reizer et al., 2022) and is generally considered to be a counterproductive behavior that can occur during school hours. Taking into consideration the influence of stressors during theoretical and practical classes in vocational training, which can lead to lower academic performance and dropout (Lizarte and Gijón, 2022a,b), we will focus on how stressors are related to cyberloafing and smartphone addiction and maladaptation as mediating variables.

The relevance of the article presented is based on the scarcity of previous studies that focus on higher vocational training students, due to the fragmentation of these teachings in very varied professional areas (professional families in their denomination in Spain), which has been highlighted by studies such as the one carried out by the Bankia Dual Foundation for the period 2005–2017 or the recent review by Toepper et al. (2022), which points to the probable causes of this scarce research in higher VET, which may also be due to the difficulty of transferring their results to general recommendations to be applied in education systems, due to the high contextualization of these studies.

### 1.1 Cyberloafing in the classroom

It is a proven fact that technological innovations (multimedia applications, web technologies and next-generation educational applications) enable courses to be delivered more effectively and interactively (Ersoy et al., 2016; Johnson et al., 2023). However, misuse of the internet in the classroom can lead to distraction and disconnection from the lesson the teacher is teaching (Wu and Xie, 2018; Flanigan and Babchuk, 2022). In relation to the above statement, accessing applications via the internet in the classroom can lead to cyberloafing in students (Baturay and Toker, 2015; Akgün, 2020), leading in some cases to lower academic performance, decreased motivation and discipline problems (Junco and Cotten, 2012; Farivar and Richardson, 2018; Gökçearslan et al., 2018). However, some research considers cyberloafing to have certain positive effects, such as stress reduction and relaxation during work sessions and the completion of large amounts of work tasks in the workplace (Yildiz and Saritepeci, 2019). The problem that arises in practice is to be able to quantify the negative or positive balance of cyberloafing for learning, both on a general and individualized level, as well as to develop teaching strategies for the management of cyberloafing as a didactic tool, although this will probably be closely related to the teaching style of the teacher and the context of each subject and educational stage.

### 1.2 Stress in der Berufsausbildung

**Figure 1:**
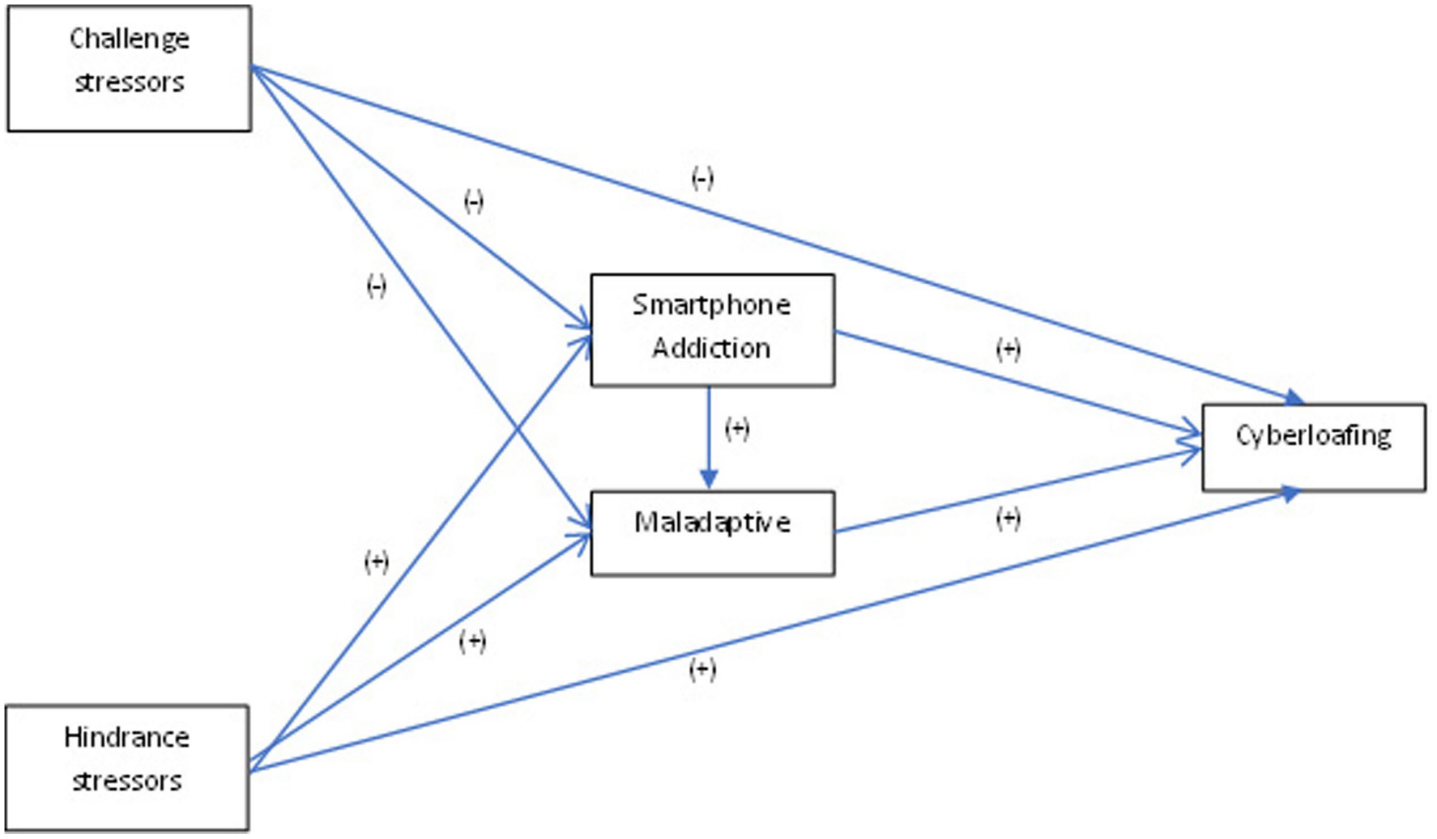
Hypothesized model of relationships among stressors, smartphone addiction, Maladaptive, and cyberloafing.

In relation to stress, the change involved in the transition from secondary school to vocational training has been studied by a number of authors (Steinmann, 2005; Pusceddu, 2016; Hösli et al., 2018; Böhn and Deutscher, 2022). It has become clear that for many students, this new stage can be more sensitive to stress, due to the cognitive, affective, biological and social changes that occur at this stage of life. Vocational trainees have to fulfil the role of a student during theoretical lessons and the new expectations and demands of the company during the traineeship (Berweger et al., 2013; Leu, 2014). For this reason, it is often more time-consuming for vocational students to regulate their stress in certain academic situations during the internship period (Schulten and Wussler, 2013). The use of different coping strategies will explain individual differences in relation to adapting to the stress required by these new educational demands (Hernández et al., 2023). See Figure 1 to visualise the hypothetical model of relationships that we will describe below.

### 1.3 Challenge-hindrance stressors and cyberloafing

To understand some implications of stress on cyberloafing, we can distinguish between stress perceived as an obstacle that can be overcome -challenge stress- or as a threat that can block job performance -hindrance stress- (LePine et al., 2005; Podsakoff et al., 2007; Karatepe et al., 2018; Afshari et al., 2022). In the first case -challenge-, the effects of this type of stress can help to reconstitute and boost individual growth, including high demands of work, responsibility and time pressure.

Indeed, the achievement of work objectives is related, in part, to the type of stressors that exist in the workplace and their perception. Some studies indicate that challenge stressors are associated with positive employee behaviors, with improved team performance, increased motivation and effort boosting (Kassam et al., 2009; Pearsall et al., 2009). However, obstacle stressors hinder goal achievement, role ambiguity and excessive concern for job security (Kassam et al., 2009; Pearsall et al., 2009; Mazzola and Disselhorst, 2019). Thus, challenging and hindering stressors influence learning, affecting the level of development of professional competences during studies (Lizarte, 2021; Lizarte and Gijón, 2022a). From a general perspective, stress has an adaptive and mainly psychological character that can manifest itself in an imbalance that results in the development of coping or discarding actions in the face of academic demands (Vorontsova et al., 2022), regardless of whether the learning takes place in the classroom or during internships (Gijón, 2020). If stress is severe and prolonged in students, it can reduce academic performance, decrease academic and social integration and may even increase the likelihood of dropping out of school (Amanvermez et al., 2022; Ang et al., 2022; Lizarte and Gijón, 2022a).

In certain situations, cyberloafing can be an isolating behavior (Askew et al., 2019; Tandon et al., 2021), a form of procrastination during lectures (Lowe-Calverley and Grieve, 2017), which can lead to decreased task completion satisfaction (Khansa et al., 2017) and increased emotional exhaustion (Koay and Soh, 2018). Thus, cyberloafing can be used by students as an avoidance strategy to temporarily avoid the stressful demands of certain actions in theory and practice classes. Therefore, we propose the following hypothesis:

*H1*: Obstacle stressors are positively related to cyberloafing.

Considering that challenge stressors motivate workers to exert effort and deploy their capacity to cope with work demands and goal fulfillment (Umer et al., 2022) and that obstacle stressors generate stress levels that hinder good performance and task achievement (Kassam et al., 2009; Kim and Beehr, 2020), it can be inferred that the teacher’s assessment of these situations will lead him/her to select appropriate methodologies to avoid cyberloafing as far as possible. One of the most commonly used methodologies in vocational training is project work, which could be appropriate to combat the negative effects of cyberloafing. This pedagogical approach refers to a didactic methodology by which one tries to acquire and develop competences through the realization of a project elaborated individually or in a group (Project-Based Learning). Project work enables cooperative learning and helps students to develop teamwork skills and improve the level of goal achievement (Lau et al., 2013). Thus, students could be more focused on achieving their goals, spending less time on cyberloafing. We therefore propose the hypothesis:

*H2*: Challenge stressors will be negatively related to cyberloafing.

### 1.4 Smartphone addiction and Maladaptive as mediating variables

Both challenge and obstacle stress affect academic performance (Kötter et al., 2017) and the acquisition of vocational competences during vocational studies (Lizarte and Gijón, 2022b). The impact of this ongoing stress during vocational training and the well-being of vocational students has not been sufficiently explored so far.

One of the issues of greatest interest in relation to stress, academic performance and cyberloafing is the use of mobile devices, especially the smartphone. While the use of smartphones for learning activities provides teaching opportunities, it can also have a negative impact on learning environments and processes (Wagner et al., 2012). Thus, cyberloafing behavior can lead to disengagement with class and reduced motivation (Gökçearslan et al., 2018). Cyberloafing behavior and smartphone addiction might be related as both are linked to addictive behavior (Gökçearslan et al., 2016, 2018) and avoidance of situations that can generate stress by fulfilling learning activities developed in class (Yildiz and Saritepeci, 2019). In this context, we propose the following hypothesis:

*H3*: Challenge stressors will be negatively related to smartphone addiction.

*H4*: Obstacle stressors will be positively related to smartphone addiction.

Since stress is related to cyberloafing, a reduction of stress would reduce the reasons for cyberloafing (Lim and Teo, 2005). Considering that hindrance stress increases negative emotions and cyberloafing (Koay et al., 2017), smartphone addiction could be related to cyberloafing (Gökçearslan et al., 2016, 2018). Considering that mobile phone addiction takes the form of uncontrollable use of smartphones (Park and Park 2017; Saritepeci, 2020), we could assume that students with high levels of smartphone addiction during lessons would increase levels of cyberloafing. Students with higher levels of smartphone addiction influenced by higher levels of stress are likely to be less responsive to academic demands, thus cyberloafing may increase. Two hypotheses derive from these statements:

*H5*: Challenge stressors will gain a positive indirect relationship with cyberloafing, mediated by Smartphone addiction.

*H6*: Obstacle stressors will gain a positive indirect relationship with cyberloafing, mediated by Smartphone addiction.

*H7*: Smartphone addiction will be positively related to cyberloafing.

Reactions to stressful situations are associated with Maladaptive rather than adaptive cognitive emotional regulation strategies (Kraaij et al., 2003). When people experience more stressors, they tend to develop Maladaptive cognitive emotional regulation strategies to cope with these stressors (Kraaij et al., 2003). Challenging stressors tend to favor positive behaviors and increase effort (Pearsall et al., 2009), so that self-blame, rumination, catastrophizing and blaming others (Maladaptive subscale) will have low values. However, obstacle stressors would increase the values of the Maladaptive subscale (Wallace et al., 2009; Sawhney and Michel, 2022). These facts allow us to indicate that challenge stressors could reduce the values of the Maladaptive scale and obstacle stressors would increase the values obtained when applying the Maladaptive scale (Wallace et al., 2009; Sawhney and Michel, 2022).

The use of Maladaptive emotional regulation strategies is associated with increased stress symptoms (Garnefski et al., 2001) and the use of adaptive emotional regulation strategies is associated with lower stress and higher self-esteem (Cutuli, 2014). Hence, the use of unadaptive emotional regulation strategies may increase smartphone addiction (Use this paragraph for analysis and conclusions). We propose:

*H8*: Challenging stressors will be negatively related to the Maladaptive.

*H9*: Obstacle stressors will be positively related to the Maladaptive.

*H10*: Smartphone addiction will be positively related to Maladaptive.

Based on the above, we can assume that increasing the means of access to ICT tools in theory and practice classes may promote cyberloafing behaviors (Akbulut et al., 2017; Saritepeci, 2020). Thus, considering that passive coping tends to be more Maladaptive, it could hinder growth opportunities, lower engagement and performance of students in situations that require some degree of stress for goal achievement (Wallace et al., 2009). Thus, the use of Maladaptive emotional regulation strategies in class would promote cyberloafing (Metin and Demirtepe, 2021).

We therefore propose:

*H11*: Challenge stressors will gain a positive indirect relationship with cyberloafing, mediated by the Maladaptive.

*H12*: Obstacle stressors will have a positive indirect relationship with cyberloafing, mediated by the Maladaptive.

*H13*: Maladaptive will relate positively to cyberloafing.

## 2 Method

### 2.1 Participants

The instruments were sent to 44 Secondary Schools teaching Higher Level Training Cycles in all the provinces that make up the Andalusian Autonomous Community during the 2021/22 academic year. A response of 427 questionnaires was obtained, of which 24 were excluded because they were incomplete. The final sample that participated in this study is 403 students of Higher Vocational Training, of which 46.15% are women and 53.85% are men, aged between 16 and 59 years old.

Table 1 shows the descriptive analysis of the demographic variables: in relation to the degree, 40.94% of the students are in the first year – corresponding to theory classes – while 59.06% are in the second year – work experience period in a company. As for the influence on the decision to enroll in Vocational Training, 75.68% did so because of the job opportunities, 16.63% because of the academic program, 4.47% because of friends and only 3.22% because of the possibility of participating in Erasmus+ or internationalization projects.

**Table 1:** Descriptive of socio-demographic variables.

	*n*	%
Qualification		
Theory students	165	40,94
Traineeships students	238	59,06
Sex		
Male	217	53,85
Female	186	46,15
What influenced you most in your decisión to enroll in Vocational Training?		
Job opportunities	305	75,68
Its academic program	67	16,63
My friends	18	4,47
Possibility to participate in Erasmus+ projects or internationalization projects.	13	3,22

### 2.2 Instruments

Obstacle and challenge stressors were measured using Cavanaugh et al. (2000) scale, which consists of 11 items. The obstacle stressors consist of 5 items (e.g., “Inability to clearly understand what is expected of me at work”) and the challenge stressors consist of 6 items (e.g., “Time pressures I experience”). Respondents were asked to rate the degree of stress at work for each item on a 5-point Likert scale (1 = not at all stressful, 5 = very stressful). The scores of the stress-challenge and stress-obstacle subscales were computed by averaging the items. Cronbach’s alpha value (Cronbach’s α) of the obstacle stressors was 0.83 and that of the challenge stressors was 0.85.

The Smartphone addiction scale-short version (SAS-SV) instrument by Kwon et al. (2013) is used to measure the level of smartphone addiction. The SAS-SV instrument is composed of 10 items (e.g., “Having a hard time concentrating in class while doing assignments, or while working due to smartphone use”). The questions were answered on a 6-point Likert scale, where 1 is “Strongly disagree” and 6 is “Strongly agree,” according to the self-report. Possible scores range from 10 to 60. Higher overall scores show a higher degree of smartphone addiction (Kwon et al., 2013). Cronbach’s alpha value (Cronbach’s α) for this scale was 0.92.

The Cognitive Emotional Regulation Questionnaire (CERQ) instrument was used to measure the Maladaptive subscale. The CERQ is used to measure nine types of specific cognitive skills. Self-blame, rumination, catastrophizing and blaming others are considered Maladaptive subscales. However, acceptance, positive refocusing, refocus on planning, positive reappraisal, and putting into perspective are considered adaptive subscales (Garnefski et al., 2002). The CERQ is composed of 36 items with 5-point Likert-type responses (1 = almost never to 5 = almost always). Higher scores indicate higher frequency of use of the specific cognitive strategy being measured. In our research only the Maladaptive subscale was adopted. Cronbach’s alpha value (Cronbach’s α) for this subscale was 0.93.

Lim’s (2002) 11-item scale (e.g., “visit sports-related websites”) was used to measure cyberloafing. Respondents were asked to rate their cyberloafing during school hours on a 5-point Likert scale (1 = not at all to 5 = extremely). Cronbach’s alpha value (Cronbach’s α) for this scale was 0.93.

### 2.3 Procedures

The research presented here was structured in four phases. In the first – instrument selection – the questionnaires indicated in the previous section were tailored and adapted for online application. The questionnaire included an explanation of the research, assuring the anonymity of the participants and the confidentiality of the data. In the second phase – sample design – a criterion sampling was carried out for the application of the adapted online instruments. For this purpose, a list was drawn up of educational centers offering full teaching of higher level training cycles belonging to the eight provinces that make up the autonomous community of the Junta de Andalucía during the 2021/22 academic year. Using the criterion of accessibility, several vocational training teachers were contacted to facilitate the distribution of the instruments among their students and the rest of the teachers in the participating centers. In the third phase – implementation – the instruments were applied via the online platform (end of April 2021). The average time to complete the two questionnaires was 40 min. In the fourth phase -analysis- the data were downloaded from the platform, and different descriptive statistics (mean and standard deviation) and multivariate statistics (structural equation model and multigroup analysis) were applied and the results analyzed.

### 2.4 Statistical analysis

Data were analyzed using IBM SPSS 25.0 and Amos. To understand the relationships between each construct, structural equation modeling (SEM) was used to examine causal relationships between constructs. Bootstrapping was used to demonstrate mediating effects. Finally, when comparing the differences between whether students are in theory or in practice, a multigroup invariance analysis using z-scores was conducted to test the possible moderating effect of this factor. Differences in z-score values between the constrained and unconstrained model were calculated, and structural trajectories in each subgroup were compared on the basis of the z-scores (Gaskin, 2012).

## 3 Results

Table 2 outlines the means and standard deviations, the Cronbach’s alpha reliability indices, and the correlations between the scales. In terms of internal consistency, the alpha index values were above 0.80, indicating high reliability.

**Table 2:** Means, standard deviations, reliabilities, and zero-order correlations among variables.

	Mean (SD)	Cronbach’s α	1	2	3	4	5
1. Challenge stressors	3.21 (0.77)	0.85	1				
2. Hindrance stressors	2.87 (0.62)	0.83	0.58***	1			
3. Smartphone Addiction	28.04 (9.25)	0.92	−0.33**	0.28**	1		
4. Maladaptive	2.32 (0,70)	0.94	−0.23*	0.31**	0.41***	1	
5. Cyberloafing	3.28 (0.73)	0.93	−0.55***	0.49***	0.37***	0.47**	1

*p < 0.05, **p < 0.01, ***p < 0.001.

As can be seen in Table 2, all scales correlate significantly with each other. Specifically, a negative and moderate correlation was observed between Challenge stressors and Smartphone Addiction (*r* = −0.33; *p* < 0.01); a significant negative correlation between Challenge stressors and Maladaptive, although weak (*r* = −0.23; *p* < 0.05); and a significant positive and strong correlation between Challenge stressors and Cyberloafing (*r* = − 0.55; *p* < 0.001).

On the other hand, significant positive correlations were found between Challenge stressors and Hindrance stressors of strong size (*r* = 0.58; *p* < 0.001). There was a weak positive significant correlation between Hindrance stressors and Smartphone Addiction (*r* = 0.28; *p* < 0.01); a moderate positive significant correlation between Hindrance stressors and Maladaptive (*r* = 0.31; *p* < 0.01); and a moderate positive significant correlation between Hindrance stressors and Cyberloafing (*r* = 0.49; *p* < 0.001). A significant positive correlation was observed between Smartphone Addiction and moderate-sized Maladaptine (*r* = 0.41; *p* < 0.001); a significant positive correlation between Smartphone Addiction and Cyberloafing (*r* = 0.37; *p* < 0.001) was also moderate. Finally, a significant positive and moderate correlation is found between Maladaptive and Cyberloafing (*r* = 0.47; *p* < 0.01). In general terms, we can observe that the means and standard deviations in the variables are obtained around mean values in all of them: Challenge stressors (*M* = 3.21; SD = 0.77); Hindrance stressors (*M* = 2.87; SD = 0.62); Smartphone Addiction (*M* = 28.04; SD = 9.25); Maladaptive (*M* = 2.36; SD = 0.945) y Cyberloafing (*M* = 3.28; SD = 0.73).

### 3.1 Structural model testing

**Figure 2:**
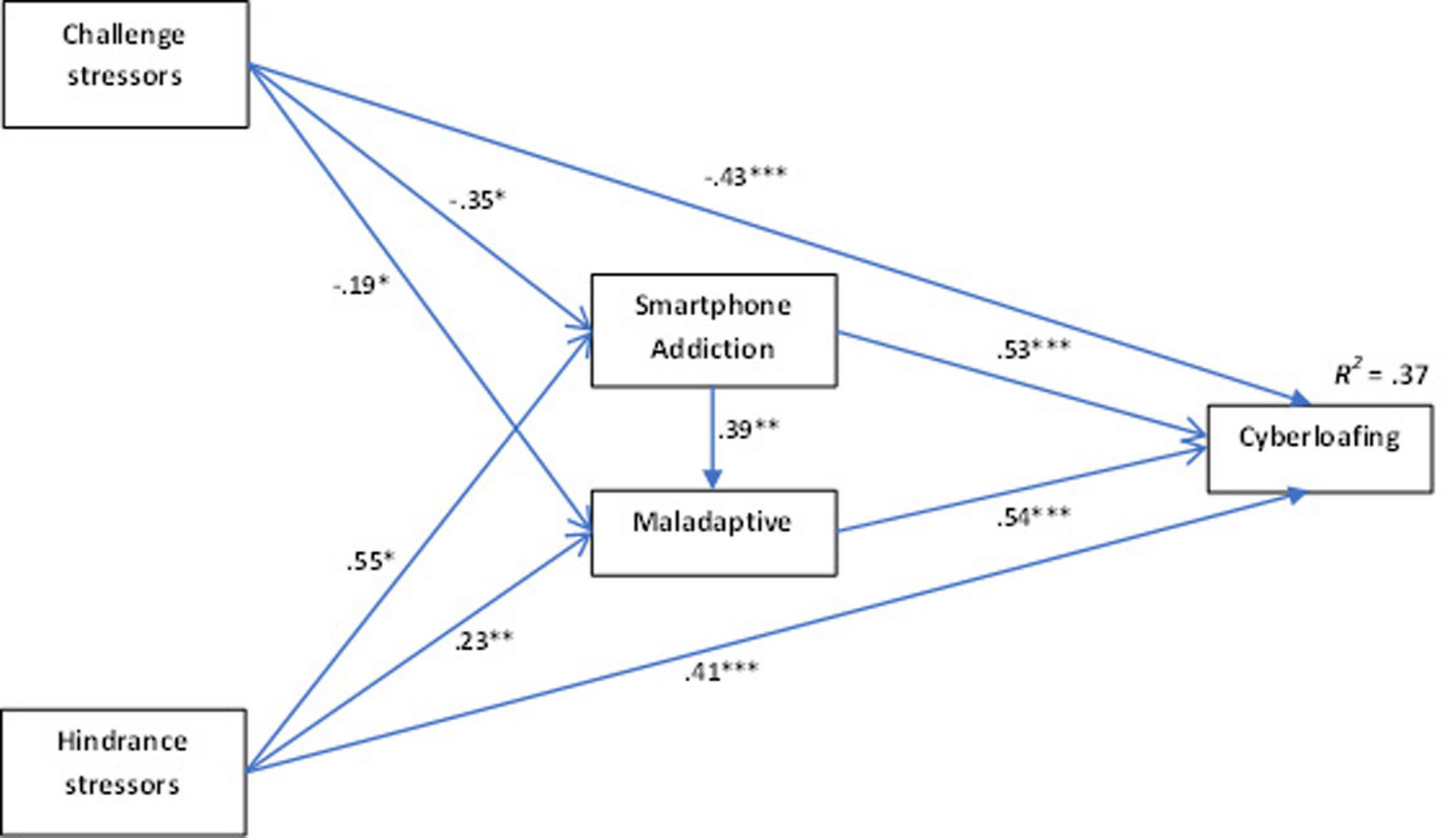
Unstandardized path coefficients for structural equation model.

The model was tested through structural equation modeling (SEM), using AMOS 26.0. The relationships between variables and the unstandardized path coefficients of the model are shown in Figure 2. Challenge stressors were negatively related to mobile addiction (*β* = −0.35, SE = 0.16, *t* = −2.15; *p* = 0.032), as were Maladaptive (*β* = −0.19, SE = 0.09, *t* = −2.01; *p* = 0.045), supporting Hypotheses 3 and 8, respectively. However, obstacle stressors were positively related to mobile phone addiction (*β* = 0.55, SE = 0.24, *t* = 2.30; *p* = 0.022) and to Maladaptive (*β* = 0.23, SE = 0.07, *t* = 3.18; *p* = 0.002) supporting hypotheses 4 and 9.

Smartphone addiction was positively related to Maladaptive (*β* = 0.39, SE = 0.14, *t* = 2.69; *p* = 0.007), thus supporting hypothesis 10.

On the other hand, both Smartphone addiction (*β* = 0.53, SE = 0.15, *t* = 3.54; *p* < 0.001) and Maladaptive (*β* = 0.54, SE = 0.24, *t* = 2.28; *p* = 0.023) were significant predictors of cyberloafing, supporting hypotheses 7 and 13. The direct pathway from challenge stressors to cyberloafing (*β* = −0.43, SE = 0.16, *t* = −6.90; *p* < 0.001) was significantly negative, thus not supporting hypothesis 2, while the direct route from obstacle stressors to cyberloafing (*β* = 0.41, SE = 0.07, *t* = 6.17; *p* < 0.001) was significantly positive, thus supporting hypothesis 1.

The fact that the direct effects of challenge and hindrance stressors on mobile addiction and cyberloafing were significant supported the possibility of an indirect effect. Bias-corrected bootstrapping was used to test for an indirect effect (Preacher and Hayes, 2008; Hayes et al., 2011). The results indicate that mobile phone addiction mediates the relationship between stressors and cyberloafing. The indirect effects of challenge and hindrance stressors on cyberloafing through mobile addiction were significant (for challenge stressors, indirect effect = −0.19, 95% CI [0.03, 0.32] supporting hypothesis 5; for hindrance stressors, indirect effect = 0.29, 95% CI [0.07, 0.43] supporting hypothesis 6). Similarly, the fact that the significant direct effects of challenge and hindrance stressors on Maladaptive and Maladaptive on cyberloafing also suggested the possibility of an indirect effect. Thus, the Maladaptive measured the relationship between stressors and cyberloafing. The indirect effects of challenge and hindrance stressors on cyberloafing via the Maladaptive were significant (for challenge stressors, indirect effect = −0.10, 95% CI [0.01, 0.21] supporting hypothesis 11; for hindrance stressors, indirect effect = 0.12, 95% CI [0.01, 0.23] supporting hypothesis 12).

### 3.2 Multigroup analysis

Multigroup analysis was performed to compare the differences in the coefficients of the trajectories according to whether the students are in theory or in practice, using the z-score comparison (Table 3). The result reveals that the relationship between Maladaptive and cyberloafing is positive in students who are in the theoretical part while in students who are in the practical part this relationship is negative, this difference being statistically significant.

**Table 3:** Multi-group analysis regression coefficients.

	Grupo		
	Teoría	Práctica	z-score
CS--->CYB	−0.37***	−0.40***	−0.57
CS--->SMA	−0.31*	−0.29*	0.32
CS--->MAL	−0.17*	−0.15*	0.29
HS--->CYB	0.35**	0.37***	0.26
HS--->SMA	0.51*	0.49*	−0.27
HS--->MAL	0.15***	0.18**	0.43
SMA--->CYB	0.47***	0.44***	−0.44
MAL--->CYB	0.41**	−0.18**	−5.36***
SMA--->MAL	0.32**	0.34**	−0.29

CS, Challenge stressors; HS, Hindrance stressors; SMA, Smartphone Addiction; MAL, Maladaptive; CYB, Cyberloafing. *p < 0.05, **p < 0.01, ***p < 0.001. *z*-score: critical ratio of differences between parameters.

## 4 Discussion and conclusions

With this research we have been able to analyze the relationship between challenge stressors, obstacle stressors, smartphone addiction, maladaptation and Cyberloafing in higher vocational training students. The mediating role of smartphone addiction and maladaptation in the relationship between challenge-obstacle stressors and Cyberloafing was analyzed. Findings suggest that students’ challenge stressors do not increase Cyberloafing, allowing them to cope with academic demands and work challenges during the theory and internship period. In contrast, obstacle stressors generate stressful situations that impair the acquisition of academic goals and the development of academic skills. In our research we observed that in vocational students, challenge-obstacle stressors have a disparate influence on cyberloafing.

Evidence was obtained that challenge stressors are negatively related to Smartphone addiction, taking into consideration that there are several methodologies that involve the use of Smartphones as a didactic resource, and smartphone addiction is related to cyberloafing. Challenge stressors were negatively related to Smartphone addiction (3). Furthermore, challenge stressors obtain a positive indirect relationship with cyberloafing, mediated by Smartphone addiction (5). The findings allow us to suggest that challenge stressors have a similar behavior to that found in studies conducted with employees (Agarwal and Avey, 2020; Zhou et al., 2021); thus, we could suppose that this type of stress would allow reinforcing the commitment and effort to meet academic and work challenges, reducing the levels of Cyberloafing (Kunzelmann and Rigotti, 2021).

The results of our research showed that, unlike challenge stressors, obstacle stressors are positively related to cyberloafing (h4), indicating that this type of stress causes an increase in cyberloafing as a temporary avoidance response to certain academic demands. Obstacle stressors have an indirect positive relationship with cyberloafing, mediated by smartphone addiction (6). This could lead to reduced academic performance and low academic and social integration. It is well known that smartphones are changing the system of relationships, lifestyles and ways of learning (Karaoglan Yilmaz et al., 2023). However, incorrect use can have a negative impact on the learning process (Wagner et al., 2012). Thus, addictive behavior and avoidance of stressful obstacle situations lead to increased levels of Smartphone addiction and Cyberloafing. As a result of this finding, it is necessary to regulate didactic planning so that the use of smartphones does not promote addiction. It must be considered that the dependence on smartphones has increased in the school environment. The smartphone has become a working tool for both teacher and pupil, and its use has become a commonly accepted habit for the teacher’s decision making in monitoring academic tasks. All this leads us to believe that the learner will become increasingly dependent on smartphones for school work. It is clear that educational programs on the responsible use of mobile phones need to continue to be implemented for students in stages prior to higher vocational education.

Our findings indicate that challenging stressors are negatively related to Maladaptive, as they tend to favor positive behaviors and effort. This relationship indicates a reduction in the values of self-blame, rumination, catastrophizing and blaming others. The same is not true for hindering stressors, increasing the use of Maladaptive emotional regulation strategies. Maladaptation leads to a further strengthening of smartphone addiction. As a result of our research, we observed that Maladaptive is higher when high values of Cyberloafing are registered; therefore, the use of Maladaptive emotional regulation strategies corresponds to avoidance behaviors that could favor Cyberloafing. Another important finding in this study, through multigrade analysis, is that we obtained a significant negative relationship between Maladaptive and Cyberloafing in internship students: higher levels of Maladaptive lower levels of Cyberloafing. During the internship period, students perceive the challenges differently than in theory class.

Thus, the perception that internship students have of the academic and work challenges or demands favor positive behavior and effort. We should bear in mind, firstly, that motivation at this final point of training may be of great importance, as the student will present a solid state of mind, which would allow a higher level of adaptation to achieve the academic objectives in a more effective and efficient way. Secondly, related to the previous statement, we can indicate that the vocational training student has chosen this training pathway oriented toward a more immediate employment opportunity, which maximizes the relationship between academic effort and the result of professional insertion. This choice is the majority of all students enrolled in higher vocational training. Despite what has been indicated in relation to trainees, some studies carried out with these students during the pandemic have shown the existence of maladjustment in an irregular way, with new forms of maladjustment appearing that had not previously been clearly established, such as the practice of being on the phone and ignoring those around them (phubbing).

Finally, we can indicate that our research also allows for a number of practical reflections on challenging stressors, which could mobilize the development of academic and professional competences. It should be noted that, while this type of stress provokes positive behaviors, it would also encourage innovative behavior in challenging situations (Ren and Zhang, 2015). These innovative behaviors can relate to the learner, the teacher, the educational program and the organization of the school institution. As for the student, learning to manage stressful situations – i.e. developing soft skills related to stress management – can be a key element for academic success and be projected as an added value for their insertion into the world of work. Related to this learning, the development in the classroom of activities that put students in the situation of having to face challenging stressors and regulate them, will allow the teacher to promote competences related to stress management. This should take the form of the design and application of activities whose learning outcomes include the aforementioned soft competences. Finally, the very organization of spaces and times in the educational institution can also be oriented toward the development of stress management skills, with spaces for relaxation or the development of actions that include activities such as Dog Assisted Therapy in pet-friendly institutions, to reduce stress at specific times, such as exam periods.

### 4.1 Limitations and options for future research

The study has several limitations. Firstly, the sample consists of higher vocational students from a limited number of vocational families. It will be necessary in the future to expand the number of vocational families in order to carry out comparative analyses. In addition, research at other levels of vocational education, such as intermediate vocational training, may give a more complete picture of the role of stressors in academic success. An additional limitation may derive from the timing of the study – during the pandemic – when personal and academic relationships were disrupted by the excessive use of smartphones, due to confinement and virtual learning. At the present time, the relationship system may have changed with the return to normality, with personal relationships recovering in the face of mobile device use. However, this needs to be investigated, to find out whether the pandemic has been a break in the normal development of educational programs or, on the contrary, has substantially changed the system of relationships, teaching methodologies and modes of learning, as well as its influence on levels of academic stress and its management.

## Data availability statement

The datasets presented in this article are not readily available because the full dataset is not available for this study. Contact the first (EL) or last author (JG) for access to specific variables. Requests to access the datasets should be directed to josegp@ugr.es.

## Ethics statement

The studies involving humans were approved by the Ethics Committee in Human Research of the University of Granada were followed 2758CEIH2022. The studies were conducted in accordance with the local legislation and institutional requirements. Written informed consent for participation was not required from the participants or the participants’ legal guardians/next of kin in accordance with the national legislation and institutional requirements.

## Author contributions

EL: Conceptualization, Formal analysis, Investigation, Methodology, Project administration, Writing – original draft. MKG: Conceptualization, Investigation, Supervision, Validation, Visualization, Writing – review & editing. MCG: Conceptualization, Data curation, Project administration, Visualization, Writing – review & editing. JG: Conceptualization, Investigation, Software, Validation, Visualization, Writing – review & editing.
